# The Pathway Building Technique in Implementation Research Using Mixed Methods Design

**DOI:** 10.1177/08445621231213432

**Published:** 2023-11-09

**Authors:** Ahtisham Younas, Caroline Porr, Joy Maddigan, Julia E Moore, Pablo Navarro, Dean Whitehead

**Affiliations:** 1 Assistant Professor, Faculty of Nursing, 7512 Memorial University of Newfoundland, St. John's, Canada; 2 Former Associate Professor, Faculty of Nursing, 7512 Memorial University of Newfoundland, St. John's, Canada; 3 Associate Professor, Faculty of Nursing, 7512 Memorial University of Newfoundland, St. John's, Canada; 4 Director, The Center of Implementation, Toronto, Canada; 5 Senior Research Officer, The Newfoundland and Labrador Centre for Applied Health Research, St. John's, Newfoundland, Canada; 6 Senior Lecturer, Institute of Health and Wellbeing, 1458 Federation University Australia, Ballarat, Australia

**Keywords:** Building technique, data integration, implementation science, mixed methods research

## Abstract

**Background:**

Data integration refers to combining quantitative and qualitative data in mixed methods. It can be achieved through several integration procedures. The building integration procedure can be used for developing quantitative instruments by integrating data from the qualitative phase. There are limited examples of data integration using the building procedure in mixed methods and implementation science.

**Purpose:**

The purpose of this article is to illustrate how the pathway building technique can be used to integrate data in mixed methods research through concurrent use of implementation science models and frameworks.

**Methods:**

This two pathway building technique was developed based on a mixed methods implementation project of developing implementation strategies to promote compassionate nursing care of complex patients.

**Results:**

The first pathway is the integration of qualitative data from the first phase of mixed methods study with implementation models and frameworks to create a quantitative instrument (i.e., a Q-sort survey) for the subsequent phase. The second pathway is the operationalization of the Q-sort survey results (i.e., implementation strategies) using an implementation science specification framework.

**Conclusion:**

The pathway technique is valuable for mixed methods research and implementation science as it offers a theory-based innovative method to tackle integration challenge.

## Introduction

Mixed methods research (MMR) is an iterative methodology that involves the integration of qualitative and quantitative paradigms and methods for developing a comprehensive understanding of research phenomena that cannot be fully achieved with qualitative and quantitative methods, alone (Bazeley, 2018; Creswell & Plano Clark, 2018). MMR has emerged as an approach to guide researchers across health sciences, nursing, midwifery, management, educational psychology, social sciences, and education (Lopez-Fernandez & Molina-Azorín, 2011). The extensive use of MMR across disciplines is due to its strength in integrating diverse and sometimes opposing paradigms and methods (Creswell & Plano Clark, 2018; Plano Clark & Ivankova, 2016). The application of MMR has been expanded to translational research (Ivankova et al., 2018; Meister, 2018) when seeking to apply pre-clinical research to inform the conduct of human trials, and for the adoption of research for practice and policymaking (Woolf, 2008).

Translational research is referred to as Knowledge Translation (KT). KT is a dynamic and iterative process comprising synthesis, dissemination, exchange, and ethically sound application of knowledge to improve the health of Canadians [individuals], to provide more effective health services and products, and to strengthen the health care system (Canadian Institutes of Health Research, 2016, p. 2). Implementation science (IS) can be considered a subset of KT and is defined as “the scientific study of methods to promote the systematic uptake of research findings and other evidence-based practices into routine practice, and, hence, to improve the quality and effectiveness of health services” (Eccles & Mittman, 2006, p. 1). Implementation scientists commonly uses MMR designs along with behavior change theories and frameworks to: a) identify factors affecting change, b) assess the quality and uptake of evidence-based practice guidelines, and c) design and evaluate implementation strategies for change (Bauer et al., 2015; Meister, 2018; Palinkas et al., 2011).

During any implementation research project, developing an adequate and comprehensive understanding of individual and group behaviors and social and health processes requires qualitative and quantitative approaches (Bazeley, 2012). Integrating findings from qualitative and quantitative approaches can further enhance understanding (Creswell & Plano Clark, 2018). Therefore, integration is the cornerstone of MMR because it contributes to the generation of robust and plausible knowledge and understanding of a given phenomenon (Bazeley, 2018; Creswell & Plano Clark, 2018) or intended behavior change (Palinkas et al., 2011; Younas, Pedersen & Tayaben, 2022). Integration should occur at the theoretical and empirical levels because it involves more than merely assembling, combining, and aggregating qualitative and quantitative data (Tunarosa & Glynn, 2017).

Integration can be accomplished by using several types of integration procedures such as connecting, merging, embedding, building (Fetters et al., 2013), threading (Moran-Ellis et al., 2006) exploring, generating a hypothesis, initiating, comparing, constructing a case, expanding, diffracting, explaining, corroborating, and enhancing (Fetters, 2019; Younas & Durante, 2022). The integration procedures can be employed under broad methods of analysis, namely, sequential, complementary, and linking methods (Bazeley, 2018). The sequential method enables development of tools, variables, programs, interventions, and the generation, testing, and evaluation of theories and models. The complementary method allows for comparing, merging, and contrasting different data sources to compare datasets. The linking method enables development of a comparative (examining differences in research findings across subgroups such as age, gender, and role), relational linkages (identifying changes in patterns and aspects of a phenomenon across cases in relation to the overarching research purpose) for enriched understanding of the phenomenon (Bazeley, 2018). Implementation scientists using MMR designs employ several integration methods and techniques but there are few, relevant and practical examples. For example, qualitative and quantitative data are combined in MMR implementation studies using merging integration procedure at the completion of qualitative and quantitative phases to develop a comprehensive understanding of a phenomenon or complex implementation issue from multiple stakeholders’ perspective. Especially needed are worked examples to demonstrate threading and building techniques (Palinkas et al., 2011). Compared to merging and connecting integration procedure, these two techniques can be complex because these are implemented during a mixed methods study rather than at the completion (Fetters, 2019). Inadequate integration during the mixed methods implementation research can affect the rigor and quality of integration in research, the developed outputs, outcomes, and the process employed during the project (Palinkas & Cooper, 2017). Therefore, this methodological manuscript focuses on the building technique which entails development of data collection instruments, tools, methods or interventions for the quantitative phase based on the findings of the first phase.

## Purpose

The purpose of this paper is to illustrate a pathway building technique for mixed methods implementation research. Pathway One is the process that can be used to integrate qualitative data to develop a quantitative instrument. Pathway Two is the process that can be used to operationalize the results of a quantitative method or in this case a data collection instrument. We illustrate how the pathway building technique can be used to integrate data in MMR designs through concurrent use of IS theories and frameworks. Illustrations are drawn from an exploratory sequential MMR study focused on the selection of relevant implementation strategies that would positively influence compassionate nursing care of complex patients.

## Overview of the compassionate nursing care mixed methods research study

This methodological paper is drawn from a larger mixed methods study of compassionate nursing care for complex patients (Younas et al., 2022a; Younas et al., 2022b). The overall purpose of this study was to understand the barriers to compassionate nursing care of complex patients and then develop implementation strategies that would overcome barriers. An exploratory sequential three-phase MMR study (Creswell & Plano Clark, 2018) was conducted. Ethical Approval was obtained from the The Health Research Ethics Board (HREB) of Newfoundland and Labrador (Approval Number# 2020.255).

Phase 1 was the qualitative component during which participants were interviewed about the nature of compassionate nursing care when attending to complex patients. The participants were recruited from community settings in Newfoundland and Labrador using posters, flyers, social media and through reaching out to community support organizations (e.g., The Gathering Place and Home Support Organizations) caring for complex patients (i.e., had multimorbities, and, or, physical and mental health issues, and, or, substance abuse, and are often impacted by sociocultural factors). The participants (*n* = 23) shared their experiences as complex patients in tertiary care centers in eastern Canada. Of 23 individuals, 19 were homeless and were living in a community shelter. Both virtual and in-person socially distanced interviews, as per participants choice, were conducted in a Downtown Shelter. During these interviews, barriers to compassionate nursing care were understood from the perspective of participants with experience as complex patients for two reasons. Initially, the purpose was to explore barriers to compassionate nursing care from the perspective of both patients and nurses. However, due to the pandemic interviews with nurses working in the hospitals were not possible therefore; only individuals with complex health issues were interviewed.

During Phase 2 (instrument development) the pathway building technique was the integration procedure used to compile a list of implementation strategies for a data collection instrument (i.e., the Q-sort survey which asks participants to rank various statements about implementation strategies in terms of their relative importance to promote compassionate behaviors of nurses) (See Supplemental Online File). Phase 3 (the quantitative component) involved distributing the Q-sort survey to nurses, nurse managers, health care administrators, policymakers and compassion care experts. Before the actual survey, the Q-sort survey was piloted with five respondents to assess feasibility, readability, and potential challenges. Then, anonymous surveys were distributed via QMethod software. Completion and return of the survey were considered research consent. In total, 105 potential respondents were identified of which 55 responded (response rate = 38%). Out of the 55 Q-sort surveys returned, 32 were fully complete and included in the final analysis (response rate = 22.1%). The participants responded from Canada, local settings (Newfoundland), Australia, Italy, the United States, United Kingdom, and ranked 21 implementation strategies based on what they deemed most effective in addressing barriers to compassionate nursing care of complex patients.

## The pathway building technique

**Figure 1.**
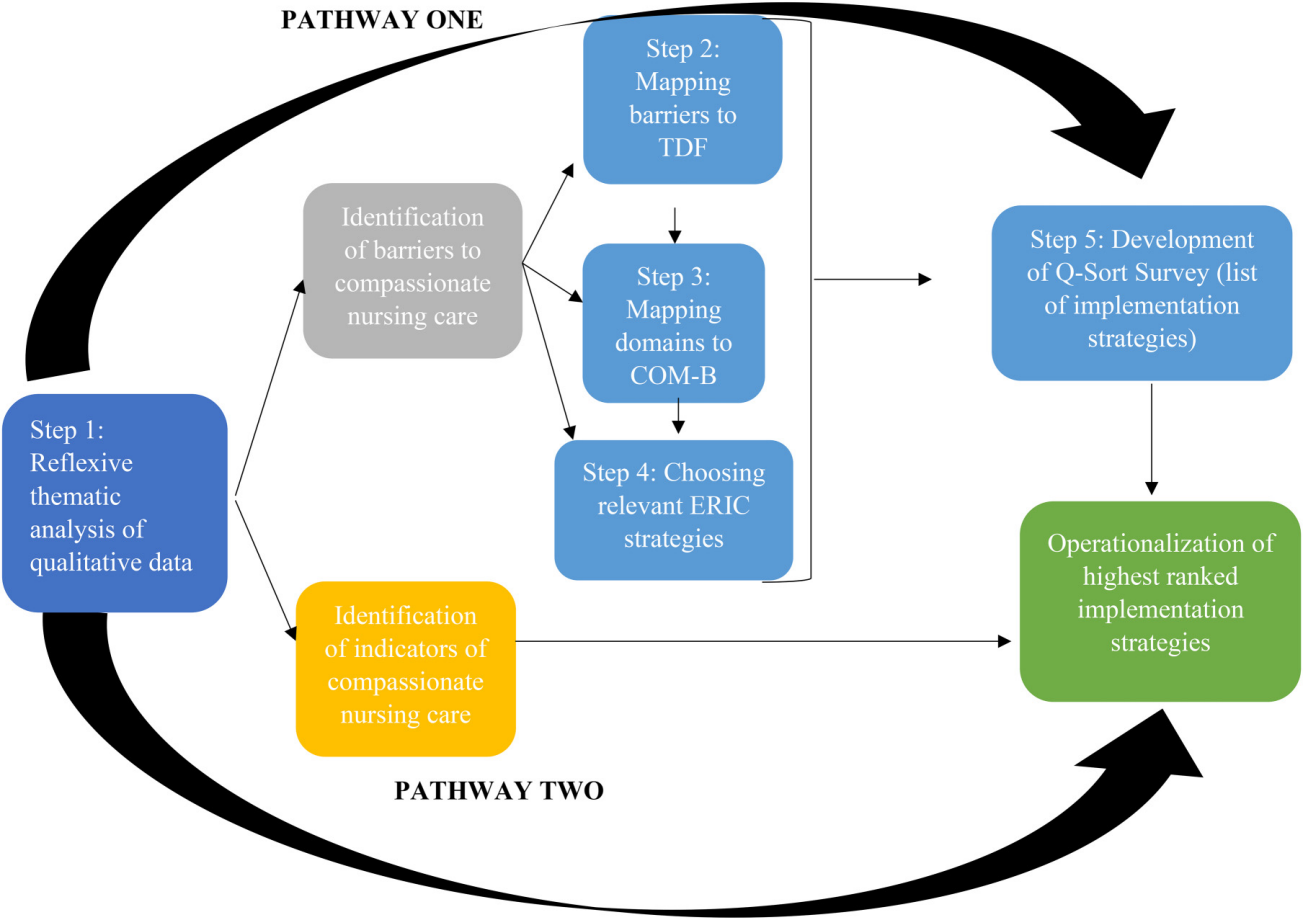
The pathway building technique. **Note:** Theoretical Domains Framework (TDF); Capability-Opportunity-Motivation-Behaviour Model (COM-B), Expert Recommended Implementation Consensus (ERIC).

The pathway approach is a novel use of the building technique. As portrayed in Figure 1, there are two pathways. Pathway One is illustrated by the process of integrating qualitative data related to the barriers to compassionate nursing care that were collected during Phase 1 of the MMR study to inform Phase 2, the development of an instrument for the quantitative phase. Pathway Two is illustrated by the process of integrating qualitative data related to the indicators of compassionate nursing care, also from Phase 1 of the MMR study, to operationalize the highest ranked implementation strategies that were the results of the Q-sort survey distributed in Phase 3.

### Pathway one: developing the Q-sort survey

Pathway One is a five-step process. Each of the five steps is described below.

#### Step 1: analysis of qualitative data

Reflexive thematic analysis of 23 qualitative interviews during Phase 1 of the MMR study resulted in contextually-relevant barriers to compassionate nursing care of complex patients. Of the several paradigmatic orientations represented by reflexive thematic analysis, for this building technique, both inductive (codes and themes are content-driven) and deductive (codes and themes are matched to an existing framework) orientations were adopted to guide the analysis that entailed: familiarization with the data, data coding, developing initial themes, reviewing themes, and defining, and naming themes (Braun & Clarke, 2019, 2021; Clarke & Braun, 2022). Based on reflexive thematic analysis 16 barriers were identified: Limited knowledge about patient needs, Unrealistic patient demands and expectations, Lack of organizational supports, Lack of compassion modeling, Limited experience, Limited motivation, Lack of appreciation, Routinization of care, Underpaid, Workload, Self-care neglect, Stress and burnout, Fears related to personal safety, Interprofessional conflicts, Nurse-patient, conflicts, and Negative personal and familial experiences.

#### Step 2: mapping barriers

During this step of the process the barriers to compassionate nursing care were mapped to the Theoretical Domains Framework (TDF) (Atkins et al., 2017) to discern the intellectual, affective, and social factors influencing nursing care behaviors. The barriers are mapped to the TDF to identify the broad targeted domains for developing behavior change implementation strategies. The TDF includes 84 constructs within 14 domains (Cane et al., 2012). As a determinant framework, the TDF enables researchers to understand and describe various individual and contextual factors influencing the implementation of strategies designed to affect change in behaviors (Nilsen, 2015). The TDF is a theoretically robust framework and was chosen over other frameworks (e.g., Consolidated Framework for Implementation Research) because barriers at the provider level (e.g., nurses’ limited knowledge about patient needs) could be better identified in terms of the behaviors that should be targeted (Atkins et al., 2017; Birken et al., 2017). The 16 barriers were mapped to the appropriate domains of the TDF for a more robust analysis. The first author mapped the barriers and facilitators along with the quotes to the TDF domains, then TDF domains to the COM-B and ERIC strategies. The remaining research team discussed the mapping process with the first author and examined the mapping process to ensure that it was accurate. The 14 domains and their definitions are presented in Table 1.

**Table 1.** Barriers, TDF, and COM-B domains and integration functions.

Barriers from Reflexive Thematic Analysis	TDF Domains	COM-B Domain	Integration Functions
Limited knowledge about patient needs	Knowledge “An awareness of the existence of something” (Cane et al., 2012, p. 13).	Capability	Education
Limited motivation Greater focus on getting things done	Intentions “A conscious decision to perform a behaviour or a resolve to act in a certain way” (Cane et al., 2012, p. 14).	Motivation	
Limited experience Lack of educational preparation	Skills “An ability or proficiency acquired through practice” (Cane et al., 2012, p. 13).	Capability	Training
Lack of compassionate modeling Limited organizational support	Social influences “Those interpersonal processes that can cause individuals to change their thoughts, feelings, or behaviours” (Cane et al., 2012, p. 14).	Motivation	Enablement and Modelling
Routinization of care	Behavioural Regulation “Anything aimed at managing or changing objectively observed or measured actions” (Cane et al., 2012, p. 13).	Capability	
Interprofessional conflicts Nurse-patient conflicts and differences	Social influences “Those interpersonal processes that can cause individuals to change their thoughts, feelings, or behaviours” (Cane et al., 2012, p. 14).	Opportunity	Enablement
Underpaid Lack of appreciation	Reinforcement “Increasing the probability of a response by arranging a dependent relationship, or contingency, between the response and a given stimulus” (Cane et al., 2012, p. 13).	Motivation	Incentivization and Modelling
Nurses’ fears about personal safety Stress and burnout Self-care neglect Negative personal and familial experiences	Emotion “A complex reaction pattern, involving experiential, behavioural, and physiological elements, by which the individual attempts to deal with a personally significant matter or event” (Cane et al., 2012, p. 14).	Motivation	Enablement and Modelling
Workload Non-supportive organizational mechanisms Negative patient behaviors Unrealistic patient demands and expectations	Environmental Context and Resources “Any circumstance of a person's situation or environment that discourages or encourages the development of skills and abilities, independence, social competence, and adaptive behaviour” (Cane et al., 2012, p. 14).	Opportunity	Environment Restructuring & Training

Reproduced and Adapted from Younas, Pedersen, & Inayat (2022) with the kind permission of Wiley & Sons.

Table is originally published in Journal of Nursing Scholarship

This mapping process was repeated until all the barriers were mapped to the TDF domains. For example, the barriers “workload,” “negative patient behaviours,” “unrealistic patient demands and expectations,” and “lack of organizational supports,” were consistent with the *environmental context and resources* domain that is defined as “any circumstance of a person's situation or environment that discourages or encourages the development of skills and abilities, independence, social competence and adaptive behaviour” (Cane et al., 2012, p. 12). Interview participants informed that sometimes complex patients and their family members could be verbally abusive toward nurses and not treat them with respect. They could also be dismissive of the challenges that nurses face in the health care setting. This description is consistent with the *environmental context and resources* domain, hence; was mapped under this domain. The complete list of barriers mapped to TDF domains is presented in Table 1.

#### Step 3: mapping relevant theoretical domains

The TDF enables in identifying the broad targeted domains and integration functions for behvaiour change. However, to identify for specific implementation functions, COM-B theoretical framework was used. The COM-B theoretical model help in diagnosing the overarching behavior classification under three domains namely, “motivation,” “capability,” and, or, “opportunity” (Michie et al., 2008, 2017). Capability refers to individuals’ having the needed knowledge and skills which demonstrates their psychological and physical capacity to perform in a particular task. Motivation pertains to mental processes energizing and directing individuals behavior including intentional and analytical decision making to perform a particular task. Finally, opportunity refers to all the external circumstances and determinants that can positively or negatively prompt engagement in certain behaviors or tasks (Michie et al., 2011).

The COM-B theoretical model enables identification of broader integration functions that are likely to be appropriate for a given context and a specific target population (Michie et al., 2008, 2011). Under these three classifications there are nine integration functions (or interventions): Education (i.e., increasing knowledge), Persuasion (i.e., using communication for promoting behavior change), Incentivization (i.e., offering rewards), Coercion (i.e., establishing probability of punishment or cost), Training (i.e., teaching skills), Restriction (i.e., reducing the opportunity to engage in a non desired competing behvaiours), Environmental Restructuring (i.e., altering the physical, social, or organizational context), Modelling (i.e., offering rewards), and Enablement (i.e., providing means to prompt behvaiour change capability) (Michie et al., 2011). For example, the *environmental context and resources* theoretical domain fell under “opportunity” when mapped to the COM-B theoretical model. The potential integration functions that would target the barriers under the *environmental context and resources* domain were Enablement, Environmental Restructuring, and Training. The integration functions relevant to the remaining barriers included Modelling, Education, and Incentivization. The mapped barriers, TDF domains, and COM-B integration functions are presented in Table 1.

In addition to sharing perspectives about barriers, interview participants also offered recommendations how to improve compassionate nursing care delivery. The participants suggested education and training focused on compassionate care, health and social needs of complex patients, establishing therapeutic rapport with patients, holistic care, and reflective practice. They also recommended organizational changes in institutional care mandates (e.g., compassionate care), interdisciplinary teamwork, hiring policies, and, changes to address health care budgets, recruitment and retainment practices, salary increases, and staffing issues. Based on these suggestions and recommendations, Training and Environmental Restructuring were selected as the most pertinent integration functions to address the barriers mapped to the *environmental context and resources* domain.

#### Step 4: choosing implementation strategies

To develop more concrete implementation strategies, the Expert Recommendations for Implementing Change (ERIC) guidelines (Powell et al., 2015) were used. The COM-B model provides a diagnostic domain for implementation, but ERIC guidelines offer more concrete and operationalizable implementation strategies for each behavioral domain. Therefore, at this step in Pathway One, for each integration function identified, the key implementation strategies were selected from the ERIC strategies. Three team members worked together to select the most appropriate strategies to address the barriers and relevant integration functions. The definition of each strategy was reviewed and potential strengths and limitations were reviewed before selecting the strategy for inclusion in the Q-Sort survey.

The ERIC strategies are derived from a three-round Delphi study of 71 global implementation scientists and experts, offering 73 concrete implementation strategies that can be used alone or in combination. Some of the examples of the implementation strategies are: accessing new funding, audit and feedback, changing physical structure and equipment, centralizing technical assistance, creating new clinical teams, developing a formal implementation blueprint, academic partnerships, educational materials, and identifying and preparing champions (Powell et al., 2015).

Changing behavior can be a long, arduous, and challenging process (Braithwaite et al., 2018; May et al., 2016). No single implementation strategy can possibly address all the real and potential barriers. Hence, multiple strategies are required (Powell et al., 2019). An exhaustive list of implementation strategies was compiled during this step. For example, as displayed in Table 2, the ERIC strategies for the integration functions of Environmental Restructuring and Training included: conducting educational meetings, developing academic partnerships, distributing educational materials, shadowing other experts, providing clinical supervision, developing academic partnerships, mandating change, purposely re-examining the implementation process, altering incentives, accessing new or revisiting existing funding, staging implementation scale-up, involving executive boards, recruiting, designating, training for leadership, identifying and preparing champions, and creating learning collaboratives.

**Table 2.** Q-set of Q-sort survey of implementation strategies to promote compassionate care.

Integration Functions	ERIC Implementation Strategies
Education & Training	**Conduct educational meetings** with nurses and administration to teach about patients’ expectations of compassionate care. **Distribute educational materials (e.g., guidelines, toolkits, and manuals)** about compassionate care of complex patients. **Shadow other experts** (i.e., nurses practicing compassion towards complex patients) and reflect and apply observed compassionate behaviors Educate managers to **provide clinical supervision** to those implementing strategies for promoting compassion towards complex patients
Environment Restructuring	5. **Recruit, designate, and train** for leaders who advocate compassionate behaviors. 6. **Identify and prepare champions (i.e., frontline nurses)** who dedicate themselves to supporting and driving through implementing compassionate behaviors. 7. **Create a learning collaborative** through formation of groups or groups of provider organizations to improve the implementation of strategies increase compassionate. 8. Organizations and nursing management could **develop academic partnerships** with local colleges for revisiting curricula and developing shared trainings on compassionate care. 9. **Mandate change** by having leadership declare the priority of compassionate care and develop policies to bring in change. 10. Organizations should **purposely re-examine the implementation** of compassionate behaviors by surveying multiple stakeholders. 11. **Alter incentive** for the adoption of compassionate behaviors in care of complex patients. 12. **Access new or revisit existing funding** to facilitate the implementation of strategies to enhance the provision of compassionate care. 13. **Stage implementation scale up** by piloting small demonstration of compassionate care strategies on complex patients.
Enablement and Modelling	14. **Promote network weaving** through building upon the existing high-quality relationships within and outside the organization to promote collaborative problem-solving for fostering compassionate care of complex patients. 15. Use **facilitation** to establish a process of interactive problem solving and support to discuss nurses’ challenges and negative encounters with complex patients and their families. 16. **Conduct local consensus discussions** to address the importance of compassionate care for complex patients and whether the action plan to improve compassion is appropriate. 17. **Provide ongoing consultation** with stress experts or counselors to address nurse burnout and promote self care. 18. **Model the intended change** by demonstrating compassionate behaviors toward peers. 19. **Identify early adopters at the local sites** to learn from their experiences of compassionate care towards complex patients. 20. **Involve patients/consumers and family members** in the efforts promote compassionate care for complex patients. 21. **Organize clinician implementation team meetings** to support providers and provide them opportunities to reflect on implementing strategies for compassionate care towards complex patients.

*Note*: The bold phrases reflect the names of ERIC strategies

#### Step 5: refining survey statements

At this final step, the selected ERIC strategies became the content of the concourse and statements for the Q-sort survey. Q methodology is an approach to explore multiple perspectives from a specific group or population for understanding complex concepts. In this methodology, specialized rank type questionnaires are used to gather specific opinions on a particular topic in order recognize, elucidate, and compare multiple opinions. The selected statements in such instruments are ranked on sorting grid that is customized to the number of statements (Watts & Stenner, 2012). We used the following grid: ranked from +3 = most agreeable to −3 = most disagreeable) (See Supplemental File).

The developed Q-sort survey was aimed to explore the perspective of compassionate care experts and stakeholders about the potential strategies for enhancing compassionate care. Statements in the Q-sort survey were refined, though, to make the implementation strategies more relevant to the intended behavior change (enhancing compassionate nursing care of complex patients) and context, but deliberately kept broad so that survey stakeholders could comprehend the meaning and objectives. The content to refine the ERIC strategies was drawn from the qualitative data of individuals with complex health problems, which further demonstrates how the instrument was build from the Phase 1 qualitative findings. In total, 21 statements to address all the barriers were included in the concourse. The complete list of ERIC strategies and the relevant COM-B domains are presented in Table 2 outlining the concourse of the Q sort survey.

At the completion of Phase 3, the highest ranked implementation strategies from the Q-sort surveys were identified using Principal Component Analysis (PCA) using Varimax rotation. Unlike traditional factor analysis, in Q methodology, participant responses/data are considered the items and respondent groupings are the variables. PCA was chosen because of the absence of theoretical justification for selecting minimum number of factors (Watts & Stenner, 2012). PCA produced an initial eight-factor solution. The relevant number of factors were chosen based on Eigenvalue assessment, scree plot, parallel analysis, the percentage of variance explained by individual factors, and the cumulative variance. The cut-off for each criterion were: Eigenvalue >1.00, two or more significant factor loadings, and slope analysis in the scree plot (Guttman, 1954; Watts & Stenner, 2012), at least 5% variance explained by an individual factor, and at least 50% variance explained by all five factors (Polit & Yang, 2016). A five-factor solution was considered relevant. All five factors accounted for 64.15% of the variance with individual factor accounting for 29.18%, 12.26%, 8.88%, 7.66%, and 6.17% of the variance. The total percentage variance of 20% to 90.8% is considered appropriate in Q-sort surveys (Churruca et al., 2021). The highest ranked implementation strategies across all five factors were identified and the decisions to select the final strategies were complemented with the interview data from Phase 1 and the respondents’ comments to open-ended questions in the Q-sort survey.

### Pathway two: operationalizing the implementation strategies

The purpose of Pathway Two was to generate the content for highest ranked implementation strategies identified at the end of the Q-sort survey. Therefore, the indicators of compassionate nursing care that were identified through reflexive thematic analysis of the qualitative data became the basis for operationalizing the highest ranked implementation strategies (i.e., the Q-sort survey results). For example, modeling was identified as one of implementation strategies, to further explicate what behaviors should be modeled, their frequency, the specific actions and actors, the qualitative responses of participants in phase 1 were used.

**Figure 2.**
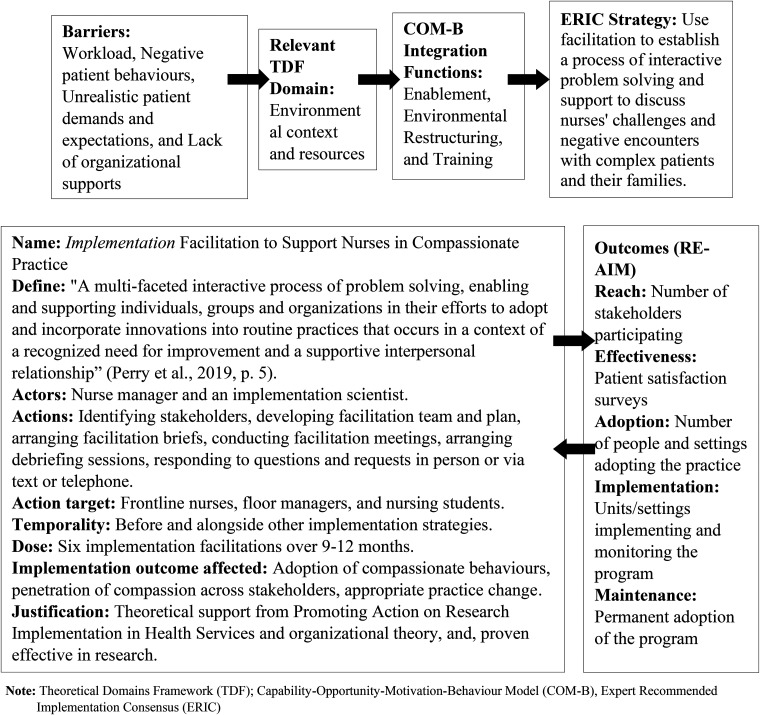
Operationalization of facilitation as an example. *Note*: Theoretical Domains Framework (TDF); Capability-Opportunity-Motivation-Behaviour Model (COM-B), Expert Recommended Implementation Consensus (ERIC).

All the selected implementation strategies were operationalized. As illustrated in Figure 2, operationalization included first defining each implementation strategy by specifying the name, conceptual definition, people implementing the strategy, the required actions, the target population, intended outcomes, and justification for using the strategy in accordance with the framework and work by Proctor et al. (2013), which required continuous reference to and consultation with the indicators of compassionate nursing care. The descriptions and words used by the participants in the qualitative interviews were used to add richness to the implementation strategies. Then measurable outcomes for all strategies were developed using the Reach, Effectiveness, Adoption, Implementation and Maintenance (RE-AIM) planning and evaluation framework (Glasgow et al., 1999).

## Discussion

The aim of this manuscript was to illustrate a novel approach to the building technique that can be useful in implementation research, exploratory sequential MMR designs. Exploratory sequential MMR designs play a significant role in designing context-specific instruments, tools, interventions, and programs (Creswell & Plano Clark, 2018) and when exploring factors that hinder implementation (Proctor et al., 2009). Therefore, MMR designs are commonly employed among implementation scientists to gain a nuanced understanding of contextual factors influencing implementation efforts (Palinkas et al., 2011; Proctor et al., 2009). One criterion of rigor in sequential MMR designs is an adequate and deliberate integration of qualitative and quantitative data which can be ensured by using the building technique (Creswell & Plano Clark, 2018; Fetters et al., 2013; Younas, Pedersen, & Inayat, 2022). The building technique explicitly links qualitative data to the quantitative phase (Fetters et al., 2013; Younas & Durante, 2022). As illustrated, the proposed pathway building technique enables researchers to make this linkage more explicit, thereby strengthening internal validity.

Generally, there is no common or concrete process how researchers are to apply the building technique because this technique is tailored to meet the study purpose and nature of the data (Fetters et al., 2013). For example, Younas et al. (2020) designed a six-step building technique to develop a data collection instrument (questionnaire) for measuring the challenges of nurse educators when teaching undergraduate nursing students. After qualitative data analysis they selected key themes and subthemes and then linked them to verbatim quotations. Then, they converted the participants’ quotations into items for the questionnaire. Unlike the pathway building technique in this manuscript, there was no underlying theory, framework, or model. Thus, the pathway building technique illustrated here is unique because it provides researchers capacity to develop instruments that are theory-driven as well as grounded in the perspectives and descriptions of participants. The proposed pathway building technique may allow researchers to use qualitative findings concurrently with theoretical frameworks and models to develop instruments or other tools for the subsequent quantitative phase.

Integration of theoretical frameworks and models has been recognized as a methodological necessity in MMR (Evans et al., 2011). However, there are limited examples of what the process exactly entails. Some researchers have elaborated using theory to guide research conceptualization, recruitment, data collection, and analysis (Alavi et al., 2018; Evans et al., 2011; Farmer et al., 2018). The pathway approach contributes to clarifying how theoretical models and frameworks can be integral to the level of data integration in MMR designs. Nevertheless, it is also important to note that researchers unfamiliar with these particular theoretical models may find the pathway approach daunting and may prefer to choose to use other implementation science frameworks which are more familiar and pertinent to their research focus. Irrespective of the kind of implementation science framework used, the pathway approach is valuable for linking theory to data and designing implementation strategies.

The pathway approach offers iterative and incremental building when qualitative findings are the basis of the quantitative phase in exploratory sequential designs (Creswell & Plano Clark, 2018). The application of this pathway approach can be further expanded for development of interventions, programs, and other tools in experimental sequential designs. For example, if qualitative interviews explored stakeholder views about certain implementation strategies, the pathway approach could incorporate design preferences as a mechanism to tailor strategies and achieve contextual relevance. Similarly, the TDF, COM-B theoretical model, and ERIC guidelines were very much integral to the pathway approach. Future research could evaluate the utility of the pathway approach using other theories, frameworks, and models in MMR designs.

The pathway approach is an innovative use of the building integration procedure for addressing the integration challenge in sequential MMR designs. The technique illustrates how qualitative findings can inform the development of a data collection instrument and then refine the quantitative results to fit the context in implementation science. Demonstrated here is robust data integration that is possible in exploratory sequential MMR designs by interconnecting theories and models from IS with MMR techniques. The pathway building technique exemplifies the complexity of integration techniques widely used in MMR designs but contributed in this manuscript are knowledge and guidelines for enabling researchers and implementation scientists to effectively achieve integration. Nurse researchers can used this pathway building approach when developing data collection instruments, interventions, or implementation strategies and their content and features in mixed methods implementation studies. Additionally, the pathways can be tailored to meet the needs and purposes of other implementation science and mixed methods based nursing projects.

## Conclusions

The iterative nature of MMR designs requires innovative ways to integrate qualitative and quantitative data to generate meaningful methods for subsequent phases of MMR studies. The pathway building technique provides an informative illustration of a novel way to build a data collection instrument from qualitative findings. The two-process pathway approach demonstrates an innovative way to incorporate empirical data, and, of equal significance is the demonstration of how theories, frameworks and models from IS can intersect with MMR design components in order to generate contextually-relevant implementation strategies.

## Supplemental Material

sj-docx-1-cjn-10.1177_08445621231213432 - Supplemental material for The Pathway Building Technique in Implementation Research Using Mixed Methods Design Click here for additional data file. Supplemental material, sj-docx-1-cjn-10.1177_08445621231213432 for The Pathway Building Technique in Implementation Research Using Mixed Methods Design by Ahtisham Younas, Caroline Porr, Joy Maddigan, Julia E Moore, Pablo Navarro and Dean Whitehead in Canadian Journal of Nursing Research
