# Supplementary material for: The Pathway Building Technique in Implementation Research Using Mixed Methods Design
Source: Can J Nurs Res. 2023 Nov 9;56(1):5–15. doi: 10.1177/08445621231213432 (PMC10804687; doi:10.1177/08445621231213432)
Supplement: sj-docx-1-cjn-10.1177_08445621231213432 - Supplemental material for The Pathway Building Technique in Implementation Research Using Mixed Methods Design [file sj-docx-1-cjn-10.1177_08445621231213432.docx]

**Q-Sort Survey**

**Strategies to Enhance Compassionate Care of Complex Patients**

**The survey is made up of three sections:**

·Demographic Information Questionnaire

·The Q-Sorting Task

·Detailed information about the development of implementation strategies

Please complete the sections **in numerical order**. The instructions are described in the relevant sections. Please follow them as carefully as possible. We hope that the process will be relatively straightforward. If, however, you have any problems or questions, please contact Ahtisham Younas by email: [**ay6133@mun.ca**](mailto:ay6133@mun.ca) or by phone: #+17099865033.

**Section 1: Demographic Information**

This section is designed to collect relevant demographic information

**Age Range: ___________ Prefer Not to Say**

**Gender: ______________ Prefer Not to Say**

**Profession: _________________________ Prefer Not to Say**

**Country of Residence: ________________ Prefer Not to Say**

If you reside in Canada or have had experience of working in Canadian heath care settings, please indicate in which part of Canada have worked _______________________________.

**Prefer Not to Say**

Have you worked in the clinical/community setting with people with complex heath care needs? Please response YES or NO. If you respond “yes”, please indicate the number of years of experience working with this patient population.

**___________________________________________________________________________**  **Prefer Not to Say**

In what capacity have you worked for complex patients in clinical/community settings? Please choose one or more of the following options:

1. Frontline Care Provider/Nurse
2. Nurse Manager
3. Case Manager
4. Hospital/Unit Administrator
5. Policymaker
6. Prefer Not to Say

Have you conducted or participated in any research projects about compassion and compassionate care? Please respond yes or no. If you respond “yes”, please indicate the number of publications on the topic of compassion and compassionate care**. ______________________________________________________________________________**

**Prefer Not to Say**

**Section 2: The Q-Sorting Task**

**For this section, the following are provided:**

·The research purpose

·The implementation strategies (i.e., 21 statements) proposed to enhance nurses’ abilities to provide compassionate care to individuals with complex health care needs (i.e., complex patients)

·The sorting distribution with ranking values

**Research Purpose**

To identify the most relevant strategies to enhance nurses’ abilities to provide compassionate care to complex patients in acute care from the perspectives of frontline nurses, nurse mangers, hospital and health administrators, and compassion research experts, and policymakers.

Complex patients are defined as “patients with multiple chronic conditions (multimorbidity), mental health issues, medication-related problems, drug-related issues, and social vulnerabilities.”

**Step By Step Instructions**

1.    The Q-sorting task requires you rank *every one* of the 21 strategies/items in the sorting distribution provided, based on the strength of your *agreement/disagreement*. The more you agree with an item, the higher the ranking you are likely to award it. The more you disagree, the lower the ranking. However, please note, that the final pattern of item rankings you produce **MUST BE THE SAME AS** the shape of the sorting distribution provided.

2.   If you look at the sorting distribution, you'll see that only ONE item can be given a ranking of +3, THREE can be given a ranking of +2, FOUR can be given a ranking of +1, and so on. Please stick to these rules. There are good reasons for the distribution, which we'll happily explain. This system is being used because it is the most effective means of capturing your perspectives for purposes of this study.

3.   Please review the list of 21 items. You now need to read each item in turn, one at a time, and divide them into three provisional ranking categories. This should be done in relation to the research purpose, so it may be as well to remind yourself of this as you go along. Category 1 should include those items with which you definitely AGREE. Put these items in a single pile. Category 2 should include those items with which you definitely DISAGREE. Put these items in a separate pile. Category 3 should include those items about which you feel INDIFFERENT, UNSURE, or which otherwise leave you with MIXED FEELINGS. These items should be placed in a separate pile directly in front of you. There are no limits to the number of items that can be placed in any of these three categories. Just be faithful to your own feelings and viewpoint.

4.    You should now have three distinct categories or piles of items. For the moment, put the items you DISAGREE with and those about which you feel INDIFFERENT to one side. Make sure you know which of these piles is which. Take the pile of items you definitely AGREE with and spread them out so that you can see them all at once. Your job is now to allocate each of these items in a ranking position at the right-hand (or agree) end of the distribution provided. Clearly, the highest rankings should be given to the items with which you agree most strongly. So, in line with the limits imposed by the distribution provided (see Step 3 above), the ONE item you find most agreeable should be awarded a ranking of +3. The next THREE most agreeable items should then be given a ranking of +2, the next FOUR would be given a ranking of +1, and so on. Keep going until ALL the AGREE items have been allocated an appropriate ranking.

**Important Notes:**

·     Don't worry if your AGREE items cross over into the negative rankings. It is not assumed that this means you disagree with the item. The ranking system is relative. When you allocate a −2 ranking, this indicates only that you probably agree with that item slightly less than the items you ranked at −1, and slightly more than those you're about to rank at −3.

·     The order in which items appear in a particular column or under a particular ranking value is irrelevant. Therefore, please don't try and order your columns!

5.    To continue sorting, you now need to follow the same procedure, but this time focusing on the pile of items you definitely DISAGREE with. Spread them out so you can see them all at once. These items will clearly be allocated ranking positions at the left-hand (or DISAGREE) end of the distribution provided. The lowest rankings should be given to the items that you disagree most strongly with. So, start at the left-hand pole of the distribution and award the ONE item you find most disagreeable a −3 ranking. The next THREE most disagreeable items would then be ranked at −2, and so on. Keep going until **ALL** the items you disagree with have been allocated an appropriate ranking.

6.    All that remains is to complete the Q-sort using the pile of items about which you feel INDIFFERENT. This is often the most difficult pile of items to sort since, by definition, you probably won't hold any strong opinions about them in either direction. In contrast, larger numbers of items can be allocated to these mid-range ranking values meaning there are comparatively few decisions to make. Again, spread the items out so you can see all of them at once and simply allocate the highest available rankings to the items with which you feel most agreement, and the lowest to those with which you feel most disagreement. Keep going until ALL your indifferent items have been allocated an appropriate ranking.

7.   Once you have completed the sorting and you have a complete Q-sort. At this stage, please have one final look at the whole thing and feel free to make any final adjustments you want to make. Check that all 21 items appear in your Q-sort and that the correct number of items has been allocated to each ranking value.

8.   Finally, please provide any detailed additional comments that you may have about the 21 items/implementation strategies. You can reflect on the strengths and limitations of these strategies, and propose any content that may be added to further refine these strategies. You can also review the next section on the development of these strategies to provide any additional insights.

| **Concourse/Q-Sort Statements** |
| --- |
| 1. Promote network weaving through building upon the existing high-quality relationships within and outside the organization to promote collaborative problem-solving for fostering compassionate care of complex patients. |
| 1. Conduct local consensus discussions to address the importance of compassionate care for complex patients and whether the action plan to improve compassion is appropriate. |
| 1. Use facilitation to establish a process of interactive problem solving and support to discuss nurses' challenges and negative encounters with complex patients and their families. |
| 1. Provide ongoing consultation with stress experts or counselors to address nurse burnout and promote self care. |
| 1. Model the intended change by demonstrating compassionate behaviours toward peers. |
| 1. Shadow other experts (i.e., nurses practicing compassion towards complex patients) and reflect and apply observed compassionate behaviours. |
| 1. Recruit, designate, and train for leaders who advocate compassionate behaviours. |
| 1. Identify and prepare champions (i.e., frontline nurses) who dedicate themselves to supporting and driving through implementation of compassionate behaviours. |
| 1. Conduct educational meetings with nurses and administration to teach about patients' expectations of compassionate care. |
| 1. Identify early adopters at the local sites to learn from their experiences of compassionate care towards complex patients. |
| 1. Distribute educational materials (e.g., guidelines, toolkits, and manuals) about compassionate care of complex patients. |
| 1. Organizations and nursing management could develop academic partnerships with local colleges for revisiting curricula and developing shared trainings on compassionate care. |
| 1. Mandate change by having leadership declare the priority of compassionate care and develop policies to bring in change. |
| 1. Involve patients/consumers and family members in efforts to promote compassionate care for complex patients. |
| 1. Organizations should purposely re-examine the implementation of compassionate behaviours by surveying multiple stakeholders. |
| 1. Offer incentives for the adoption of compassionate behaviours in the care of complex patients. |
| 1. Access new or revisit existing funding to facilitate the implementation of strategies to enhance the provision of compassionate care. |
| 1. Create a learning collaborative through formation of groups or groups of provider organizations to improve the implementation of strategies to increase compassionate care. |
| 1. Organize clinician implementation team meetings to support providers and provide them opportunities to reflect on implementing strategies for compassionate care towards complex patients. |
| 1. Stage implementation scale-up by piloting small demonstrations of strategies to enhance compassionate care of complex patients. |
| 1. Educate managers to provide clinical supervision to those implementing strategies for promoting compassionate care of complex patients. |

**Sorting Distribution with Ranking Values**

|  |  |  |  |  |  |  |
| --- | --- | --- | --- | --- | --- | --- |
|  |  |  |  |  |  |  |
|  |  |  |  |  |  |  |
|  |  |  |  |  |  |  |
|  |  |  |  |  |  |  |
| **-3** | **-2** | **-1** | **0** | **1** | **2** | **3** |

**Least Agreement** **Most Agreement**

| 1. **Please provide additional comments that you may have about the implementation strategies listed in the Q-Sort Survey.** 2. **Please share any challenges that you may have encountered during the completion of this survey.** |
| --- |

**Section 3: Development of the Implementation Strategies**

The list of 21 implementation strategies were developed from interviews with 23 individuals (and their family members) who had acute care experiences as complex patients. Based on qualitative data analysis, the barriers to the provision of compassionate care were identified:

- Limited Knowledge About Patient Needs
- Limited Experience
- Lack of Educational Preparation
- Underpaid
- Lack of Appreciation
- Limited Motivation
- Greater Focus on Getting Things Done
- Workload
- Negative Patient Behaviours
- Unrealistic Patient Demands and Expectations
- Lack of Compassion Modelling
- Lack of Organizational Supports
- Interprofessional Conflicts
- Nurse-Patient Conflicts
- Nurses’ Fears Related to Personal Safety
- Stress and Burnout
- Self-Care Neglect
- Negative Personal and Familial Experiences
- Routinization of Care

The identified barriers were mapped against the Theoretical Domains Framework (TDF) and the Capability, Opportunity, Motivation, Behaviour (COM-B) theoretical model to identify relevant domains and integration functions. Finally, broad and specific implementation strategies were selected using the ERIC guidelines (See Box 1 and Box 2). The list of 21 strategies provided for your ranking are addressing the above listed barriers and the domains and integration functions displayed in Box 1 and Box 2. This information is provided for your consideration so that you can better evaluate the relevance of the listed implementation strategies. Please let me know if you would like to have more information about the listed implementation strategies before you complete the Q-Sort Survey.

| **Box 1** | | | |
| --- | --- | --- | --- |
| **TDF Domains** | **COM-B Domain** | **Barriers/Facilitators** | **Integration Functions** |
| Knowledge | Capability | Limited knowledge about patient needs | Education |
| Intentions | Motivation | Limited motivation  Greater focus on getting things done |  |
| Skills | Capability | Limited experience  Lack of educational preparation | Training |
| Social influences | Motivation | Lack of compassion modelling  Lack of organizational supports | Enablement and Modelling |
| Behavioural Regulation | Capability | Routinization of care |  |
| Social influences | Opportunity | Interprofessional conflicts  Nurse-patient conflicts | Enablement |
| Reinforcement | Motivation | Underpaid  Lack of appreciation | Incentivization and Modelling |
| Emotion | Motivation | Nurses’ fears related to personal safety  Stress and burnout  Self-care neglect  Negative personal and familial experiences | Enablement and Modelling |
| Environmental Context and Resources | Opportunity | Workload  Lack of organizational supports | Environmental Restructuring |
| Environmental Context and Resources | Opportunity | Negative patient behaviours  Unrealistic patient demands and expectations | Training |

| **Box 2** | |
| --- | --- |
| **Integration Functions** | **ERIC Implementation Strategies** |
| Education and Training | **Conduct educational meetings:** Hold meetings targeted toward different stakeholder groups (e.g., providers, administrators, other organizational stakeholders, and community, patient/consumer, and family stakeholders) to teach them about the clinical innovation  **Develop academic partnerships:** Partner with a university or academic unit for the purposes of shared training and bringing research skills to an implementation project  **Distribute educational materials:** Distribute educational materials (including guidelines, manuals, and toolkits) in person, by mail, and/or electronically  **Shadow other experts:** Provide ways for key individuals to directly observe experienced people engage with or use the targeted practice change/innovation  **Provide clinical supervision:** Provide clinicians with ongoing supervision focusing on the innovation. Provide training for clinical supervisors who will supervise clinicians who provide the innovation |
| Environmental Restructuring | **Involve executive boards:** Involve existing governing structures (e.g., boards of directors, medical staff boards of governance) in the implementation effort, including the review of data on implementation processes  **Recruit, designate, and train for leadership:** Recruit, designate, and train leaders for the change effort  **Identify and prepare champions**: Identify and prepare individuals who dedicate themselves to supporting, marketing, and driving through an implementation, overcoming indifference or resistance that the intervention may provoke in an organization  **Create a learning collaborative:** Facilitate the formation of groups of providers or provider organizations and foster  a collaborative learning environment to improve implementation of the clinical innovation  **Develop academic partnerships:** Partner with a university or academic unit for the purposes of shared training and bringing research skills to an implementation project  **Mandate change:** Have leadership declare the priority of the innovation and their determination to have it implemented  **Purposely reexamine the implementation:** Monitor progress and adjust clinical practices and implementation strategies to  continuously improve the quality of care  **Alter incentive:** Work to incentivize the adoption and implementation of the clinical innovation  **Access new or revisit existing funding:** Access new or existing money to facilitate the implementation  **Stage implementations scale up:** Phase implementation efforts by starting with small pilots or demonstration projects and gradually move to a system wide rollout |
| Enablement and Modelling | **Promote network weaving:** Identify and build on existing high-quality working relationships and networks within and outside the organization, organizational units, teams, etc. to promote information sharing, collaborative problem-solving, and a shared vision/goal related to implementing the innovation  **Facilitation:** A process of interactive problem solving and support that occurs in a context of a recognized need for improvement and a supportive interpersonal relationship  **Recruit, designate, and train for leadership:** Recruit, designate, and train leaders for the change effort  **Conduct local consensus discussions:** Include local providers and other stakeholders in discussions that address whether the chosen problem is important and whether the clinical innovation to address it is appropriate  **Provide ongoing consultation:** Provide ongoing consultation with one or more experts in the strategies used to support implementing the innovation  **Model the intended change:** Model or simulate the change that will be implemented prior to implementation  **Identify early adopters at the local sites:** Identify early adopters at the local site to learn from their experiences with the practice innovation  **Involve patients/consumers and family members:** Engage or include patients/consumers and families in the implementation effort  **Organize clinician implementation team meetings:** Develop and support teams of clinicians who are implementing the innovation and give them protected time to reflect on the implementation effort, share lessons learned, and support one another’s learning |

**Thank you for completing this survey. Your responses are greatly appreciated.**
